# Immune-Inflammatory and Metabolic Effects of High Dose Furosemide plus Hypertonic Saline Solution (HSS) Treatment in Cirrhotic Subjects with Refractory Ascites

**DOI:** 10.1371/journal.pone.0165443

**Published:** 2016-12-12

**Authors:** Antonino Tuttolomondo, Domenico Di Raimondo, Chiara Bellia, Giuseppe Clemente, Rosaria Pecoraro, Carlo Maida, Irene Simonetta, Valerio Vassallo, Danilo Di Bona, Eliana Gulotta, Marcello Ciaccio, Antonio Pinto

**Affiliations:** 1 U.O.C di Medicina Interna e con Stroke Care, Dipartimento Biomedico di Medicina Interna e Specialistica (Di.Bi.M.I.S), Università degli Studi di Palermo (Italy); 2 Sezione di Biochimica Clinica e Medicina Molecolare Clinica, Dipartimento di Biopatologia e Biotecnologie Mediche e Forensi, Università degli Studi di Palermo U.O.C. CoreLab, Azienda Ospedaliera Universitaria Policlinico, Palermo, Italy; 3 School and Chair of Allergology, Dipartimento delle Emergenze e Trapianti d'Organo, University of Bari, Italy; 4 Dipartimento di Chirurgia Generale e d'Urgenza, Policlinico Universitario "Paolo Giaccone, University of Palermo, Italy; Universidad de Navarra, SPAIN

## Abstract

**Introduction:**

Patients with chronic liver diseases are usually thin as a result of hypermetabolism and malnutrition expressed by reduced levels of leptin and impairment of other adyponectins such as visfatin.

**Aims:**

We evaluated the metabolic and inflammatory effects of intravenous high-dose furosemide plus hypertonic saline solutions (HSS) compared with repeated paracentesis and a standard oral diuretic schedule, in patients with cirrhosis and refractory ascites.

**Methods:**

59 consecutive cirrhotic patients with refractory ascites unresponsive to outpatient treatment. Enrolled subjects were randomized to treatment with intravenous infusion of furosemide (125–250mg⁄bid) plus small volumes of HSS from the first day after admission until 3 days before discharge (Group A, n:38), or repeated paracentesis from the first day after admission until 3 days before discharge (Group B, n: 21). Plasma levels of ANP, BNP, Leptin, visfatin, IL-1β, TNF-a, IL-6 were measured before and after the two type of treatment.

**Results:**

Subjects in group A were observed to have a significant reduction of serum levels of TNF-α, IL-1β, IL-6, ANP, BNP, and visfatin, thus regarding primary efficacy endpoints, in Group A vs. Group B we observed higher Δ-TNF-α, Δ-IL-1β, Δ-IL-6, Δ-ANP, Δ-*BNP*, Δ-visfatin, Δ-Leptin at discharge.

**Discussion:**

Our findings underline the possible inflammatory and metabolic effect of saline overload correction in treatment of cirrhosis complications such as refractory ascites, suggesting a possible role of inflammatory and metabolic-nutritional variables as severity markers in these patients.

## Introduction

Cirrhosis and congestive heart failure (CHF) share an impairment of circulatory and volume homeostasis eliciting neuro-hormonal and inflammatory responses and leading to retention of sodium and water.

With regard of inflammatory response, cytokines regulate some pathologic processes in the liver, such as liver growth and regeneration, liver damage in viral liver disease, liver fibrosis in cirrhosis [1]

Furthermore, it has been reported that patients with chronic liver diseases appear usually thin as a consequence of hypermetabolic state, diminished food intake, and malnutrition. Leptin, an adipocytokine, has been reported as involved in this process [2] Other adipocytokines play an important role in lipid metabolism and liver disease progression. Visfatin a 52-kDa protein have been reported as highly expressed by liver, muscle [3] and by adipose tissue [4]

A recent study [5]reported that patients with chronic HBV infection showed higher serum levels of adiponectin and visfatin, but lower leptin levels than healthy controls, and the same authors reported a significant positive correlation between serum adipocytokine levels and liver fibrosis stages.

Another recent study [6] reported that in subjects with alcoholic cirrhosis following adjustment for fat mass, visfatin levels were significantly higher from Child-Pugh Class A to Class C.

Furthermore, leptin is strictly linked with hepatic metabolism [7] and a very recent study [8]reported a up-regulation of leptin in subjects with non-alcoholic steatohepatitis (NASH), promoting liver fibrosis by means hepatic stellate cells (HSC) via the hedgehog and the hedgehog-regulated osteopontin (OPN) pathways.

Nevertheless, to the best of our knowledge no study has addressed the effectiveness of treatment of complications of cirrhosis such as ascites and fluid overload on these inflammatory and metabolic abnormalities.

Our group in a recent clinical trial reported [9] safety and effectiveness of intravenous hypertonic saline solutions (HSS) plus high-dose furosemide compared to seriated paracentesis in subjects with cirrhosis and refractory ascites. Furthermore, our group [10] reported that high-dose furosemide plus small-volume hypertonic saline solutions (HSS) is effective on lowering natriuretic peptides and immune-inflammatory marker levels.

### Specific objective and hypothesis

On this basis, the hypothesis of our study was that the clinical effectiveness of high dose furosemide + HSS could be accomplished by parallel effects on inflammatory, natriuretic and metabolic pathways expressed by changes of cytokines, natriuretic peptides, leptin and visfatin serum levels after treatment.

On the basis of our previous own results from another clinical trial [10], we evaluated the metabolic and inflammatory effects of intravenous high-dose furosemide plus HSS compared with repeated paracentesis and a standard oral diuretic schedule, in patients with cirrhosis and refractory ascites, evaluating their effects on a panel of serum biomarkers such as some inflammatory cytokines, ANP/BNP, leptin and visfatin serum levels by means of analysis of differences of their serum levels before and after treatment with high dose furosemide + HSS.

## Materials and Methods

All consecutive cirrhotic patients presenting with ascites unresponsive to ambulatory treatment at Palermo University Hospital (*Azienda Ospedaliera Policlinico ‘Paolo Giaccone’)* who were admitted to the Internal Medicine Ward of Palermo University Hospital from December 2013 to December 2015 were offered enrolment in the study protocol after a diagnosis of ascites had been made and all potential contraindications excluded.

Refractory ascites has been defined according to the International Ascites Club criteria [11] as: (a) diuretic-resistant refractory ascites: <1.5kg ⁄week weight loss while being treated with furosemide (160mg⁄day) and spironolactone (400mg⁄day) or an equivalent dose of a loop-acting and distal-acting diuretic; or (b) diuretic-intractable refractory ascites: <1.5kg⁄week weight loss as a result of the inability to use an effective dose of diuretic because of development of diuretic-induced hyponatremia (sodium level <125mEq⁄ L), hyperkalemia (potassium level >5.5mEq⁄L), renal failure (doubling of serum creatinine or values >2.5g⁄dL) or encephalopathy; (c) previous dietary restriction of sodium between 50-66mEq ⁄day. Exclusion criteria were: non-cirrhotic ascites, congestive heart failure, acute renal failure, hepatocellular carcinoma (13), cancer, rheumatologic diseases or acute infectious diseases. The study was approved by University Policlinico P. Giaccone Ethics Committee and written informed consent was obtained for all patients. Trial registration on Clinical Trials.gov for technical problems due to the registration process has been completed in June 2016 when the researchers have yet completed the study [ClinicalTrials.gov Identifier: NCT02821377].

The authors confirm that all ongoing and related trials for this drug/intervention are registered.

The clinical trial design is based on previous own results from another clinical trial [10] conducted by our group that was not registered for technical problems linked to registration site and because the journal that at the end of the trial published it did not require trial registration. This trial is a new trial enrolling different subjects in different enrollment times and does not represent an extension of this previous trial or a follow-up study of the same patients

## Daily clinical and laboratory evaluation

### Treatment protocol (Fig 1) (S1 File, S2 File, S3 File)

**Group A:** treatment with intravenous infusion of furosemide (doses 125–250mg⁄ bid) plus small volumes of HSS (150mL 1.4–4.6% NaCl), from the first day after admission until 3 days before discharge, with water restriction and a normal sodium diet.

**Fig 1:**
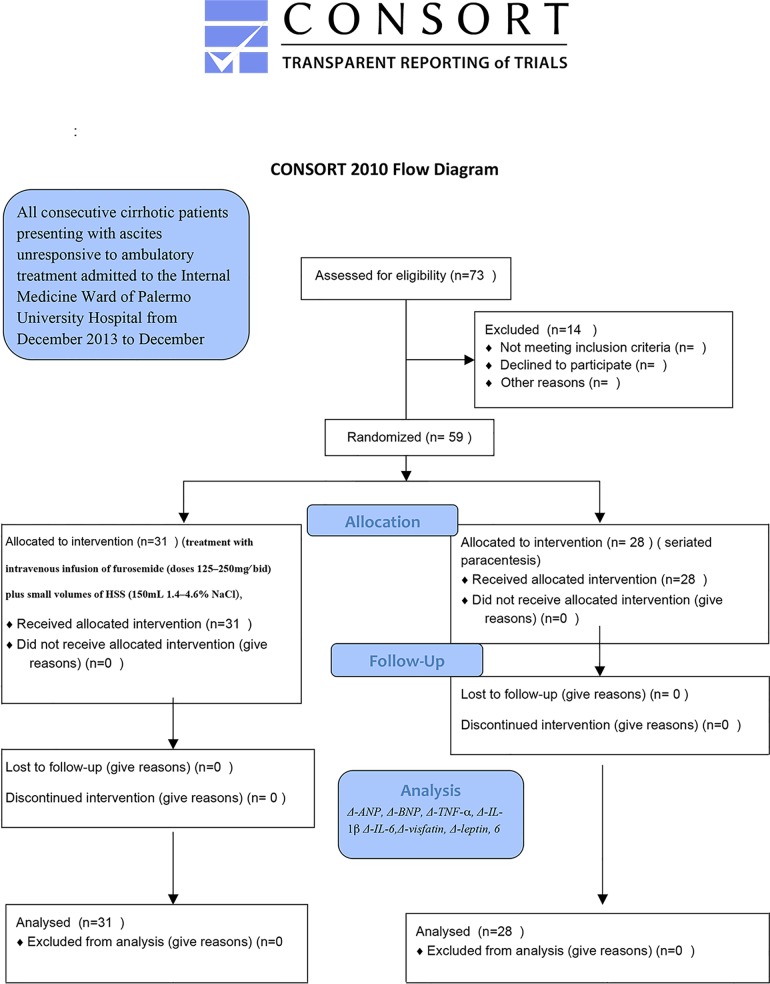
Immune-inflammatory and metabolic effects of high dose furosemide plus hypertonic saline solution (HSS) treatment in cirrhotic subjects with refractory ascites.

**Group B:** repeated paracentesis (4–6 L daily) from the first day after admission until 3 days before discharge with albumin reinfusion at a rate of 5-8g⁄ L of removed ascites. The last paracentesis (*at 3 days from admission*) was a total paracentesis (8.1±2.7L) plus *iv* albumin infusion (*8g per liter of ascitic fluid removed*) following a method previously described.

### Blood sample collection (Fig 1)

Blood samples from each subject enrolled were drawn after at least 30min of bed rest in a supine position, within 24h of admission and after 8 days of active treatment. Blood samples were centrifuged (10,000g) and the resulting supernatant was immediately frozen at -80°C until analysis was completed.

## Metabolic and immune-inflammatory biochemical evaluation

We evaluated plasma levels of ANP, BNP, Leptin, visfatin, and IL-1β, TNF-a, IL-6 were measured using a sandwich ELISA (Human IL-1β, TNF-a, IL-6 6 Diaclone). ANP and BNP plasma concentration was measured in duplicate by a solid phase sandwich immune-radiometric assay for human BNP (IRMA, ANP and BNP, Shering cis bio int). The minimum detectable concentrations for the diagnostic tests are: TNF-a: 8pg/mL; IL-1β: <1pg/mL; IL-6: <0.81pg/mL; ANP: 3.1pg/mL; BNP: 5pg/mL.

Leptin and visfatin were measured by ELISA Sandwich (leptin Mediagnostand visfatin Phoenix Pharmaceticals Inc); the minimum detectable concentration for these diagnostic teste were: leptin 0.8ng/ml; visfatin: 1.8ng/ml.

As primary efficacy endpoint we chose to evaluate the difference (Δ) between admission value and discharge value of some laboratory variables:

- *Δ-ANP (pg/ml)*: evaluated by means of the difference between ANP plasma levels at admission and ANP plasma levels at discharge.
- *Δ-BNP (*pg/ml*)*: evaluated by means of the difference between BNP plasma levels at admission and BNP plasma levels at discharge.
- *Δ-TNF-α (*pg/ml*)*: evaluated by means of the difference between *TNF-α* plasma levels at admission and *TNF-α* plasma levels at discharge.
- *Δ* IL-1β *(*pg/ml*)*: evaluated by means of the difference between IL-1β plasma levels at admission and IL-1β plasma levels at discharge.
- Δ-visfatin (ng/ml): evaluated by means of the difference between serum visfatin at admission and serum visfatin at discharge.
- Δ-Leptin (ng/ml): evaluated by means of the difference between serum leptin at admission and serum leptin at discharge.

## Statistical Analysis

Results are presented as means ± SD. Analyses of the data were performed using the unpaired Student's t test and the Mann-Whitney non-parametric test. The chi-squared test was used for comparing effectiveness endpoint differences between cases and controls. To calculate the number of patients to be enrolled, we defined as meaningful efficacy a significant difference in metabolic and immune-inflammatory variables at discharge between the two groups, with a two tailed beta error of 20% and a power of 0.80. We added 7 more patients to the estimated sample size of 40 patients to compensate for possible drop outs; the final sample, therefore, comprised 47 patients. Data were analyzed by IBM SPSS Software 22 version (IBM Corp., Armonk, NY, USA). All p-values were two-sided and p<0.05 was considered statistically significant

## Results

We recruited 73 subjects with refractory ascites (nine subjects were excluded on the basis of exclusion criteria and five patients refused to participate in the study). Fifty-nine (59 patients, 38 men and 21 women) represented the final sample. General and clinical characteristics of enrolled patients are listed on Table 1.

**Table 1:** Demographics, clinical and laboratory characteristics of subjects with refractory ascites.

	High dose furosemide +HSS	Seriate paracentesis
Number of subjects	31	28
Gender (M/F)	18/13	18/10
Age (years) (mean ±sd)	64.7 ± 10.5	62. 9 ± 8.2
SBP (mm/hg) (mean ±sd)	121±8.9	123±9.1
DBP (mm/hg) (mean ± ds)	84±4.2	80±5.6
Heart rate (beats/min) (mean ± ds)	81±6.5	79±5.7
• Etiology of cirrhosis(n/%) ○ HBV ○ HCV ○ HCV/HBV ○ Alcohol related	• • 4 (12.90) • 16 (51.61) • 5 (16.12) • 6 (19.35)	○ ○ 3 (10.71) ○ 16 (57.14) ○ 4 (14.28) ○ 5 (17.85)
• Diuretic-resistant refractory ascites, n (%) • Diuretic-intractable refractory ascites, n (%)	• 13 (41.93) • 18 (58.06)	• 16 (57.14) • 12 (42.85)
• Pre-treatment diuretics • *furosemide*, n (%) • *spironolactone*, n (%)	• • 27 (87.09) • 29 (93.54)	• • 28 (100) • 27 (96.42)
Baseline furosemide dosage (mg/day) (mean ±sd)	180.96±21.03	179.35 ±20.46
Baseline spironolactone dosage (mg/day) (mean ±sd)	429.00±53.05	423.31±53.16
Mean daily intravenous furosemide dosage (mg/day) (mean ±sd)	275.90±47.65	**-**
Esophageal varices (F1/F2/F3); n (%)	11 (35.48); 10 (32.25); 10 (32.25)	7 (25); 9 (32.14); 12 (42.85)
Bilirubin (mg/dl) (mean ±sd))	2.4±0.9	2.9±1
Albumin (g/L) (mean ±sd)	2.6±4	2.7±6
Prothrombin time (% of control) (mean ±sd)	41 ± 14	47±17
INR (mean ±sd)	1.74±1.3	1.83±1.3
serum sodium (mEq/L) (mean ±sd)	132±1.4	131±1.7
serum potassium (mEq/L) (mean ±sd)	4.1±0.8	4.4±0.3
Diuresis (ml/24 h) (mean ±sd)	850±147	825±198
Spot urinary sodium (mEq/L) (mean ±sd)	37.5 ±8.8	39.8 ±9.2
Urinary Na (mEq/24 h) (mean ±sd)	82 ± 25	79±12.4
Intrahospital deaths (n/%)	2(3.92)	4 (12.90)
Child Pugh (mean ±sd)	8.1±1.9	8.0±2.2
MELD score (mean ±sd)	21 (2.1)	20 (1.8)
The mean volume of removed ascites (mL) (mean ±sd)		4800 (1200)
Total volume of removed ascites (mL) (mean ±sd)		16400 (3400)

Demographic and clinical data are expressed as percentage (n°). Laboratory variables are expressed as mean ± ds.

SBP: systolic blood pressure; DBP; diastolic blood pressure; HR: heart rate; HBV: hepatitis B virus: HCV: Hepatitis C virus; Pre-treatment drugs: drugs used immediately prior to hospitalization or study enrolment; sd: standard deviation.

The mean age was 64±13.6 years. Patients were randomized into two groups.

Group A, 31 patients: 16 (51.61%) had Hepatitis C (HCV) cirrhosis; 4 patients (12.9%) had Hepatitis B virus (HBV) cirrhosis; five (16.12%) had combined HCV⁄HBV cirrhosis; 6 (19.35%) had alcohol cirrhosis without viral infection.

Group B, 28 patients: 16 patients (57.14%) had HCV cirrhosis; 3 patients (10.71%) had HBV cirrhosis; 4 (14.28%) had combined HCV⁄HBV cirrhosis and five (17.85%) had alcohol cirrhosis. In Group B the mean number of paracentesis performed in the whole group was 2.5±0.95 (range 1–4), the mean volume of ascites removed was 4.8±1.3 L.

Daily dosage of furosemide was defined considering urinary volume, blood pressure values and severity of ascites. The dose of HSS was determined in each patient according to these schedules: for serum Na values < 125 mEq/L, the HSS concentration was 3.5% and for serum Na values ≥ 135 mEq/L the HSS concentration varied between 1.4% and 2.4%.

The mean administered dose of furosemide in group A was 275.90±47.65 mg/die. Baseline spironolactone dosage was 429.00±53.05 mg/day in group A patients and 423.31±53.16 in group B patients (p = 0.71); baseline furosemide dosage was 180.96±21.03 mg/day in group A patients and 179.35 ±20.46 mg/day in group B patients (p = 0.56) (Table 1)

At discharge, the patients in Group A had significantly higher diuresis and sodium plasma levels and significantly lower body weight and leg edema and pleural effusion prevalence and median Child Pugh score; the median change in Child-Pugh score at discharge was significantly higher in Group A compared with Group B (1.6 vs. 0.9; P< 0.05) (Tables 2 and 3). At discharge 26 (83.87%) subjects in Group A had grade I ascites at discharge vs. 12 (42.8) subjects in Group B (p<0.001), 5 (16.12%) vs. 10 (35.71%) in group B had grade II ascites (p<0.001) whereas 0 vs. 6 (21.42%) in Group B had grade III ascites (p = 0.029).

**Table 2:** Clinical and Laboratory variables before (at admission) and after treatment with high dose furosemide + HSS (Group A) or after seriated paracentesis (Group B).

	Furosemide plus HSS (n: 31)			Seriated paracentesis (n: 28)		
	Before	After	p	Before	After	p
Number of subjects	31	31		28	28	
Weight (Kg) (mean ±sd)	76±6.6	70±6.4	<0.001	77±3.8	75.1±2.8	<0.001
Diuresis (ml/24 h) (mean ±sd)	850±147	1805±131	<0.05	825±198	950±124	0.07
creatinin (mg/dl) (mean ±ds)	1.6±0.5	1.25±0.3	0.06	1.66±0.6	1.96±0.6	0.08
• Ascites n (%) • grade I • grade II • grade III	• 12(38.70) • 10 (32.25) • 9 (29.03)	• • 26 (83.87) • 5 (16.12) • ----	• • <0.001 • <0.001 • <0.001	• • ---- • 12 (42.85) • 16 (57.14)	• • 12 (42.8) • 10 (35.71) • 6 (21.42	• • • 0.032 • <0.001
Child Pugh score (mean ±sd)	8.2	7.6	0.037	9.8	8.9	0.045
HE (n / %)	8 (15.68)	6 (11.76%)	0.82	4 (12.90)	5 (16.12)	0.78
SBP (n / %)	**-**	**-**	**-**	-	6 (19.35)	0.002
ANP (pg/ml) (mean ±sd)	32±28	22±15	<0.001	29±2.5	25±2.5	0.021
BNP (pg/ml) (mean ±sd)	13.6±3.8	7.5±3.1	<0.05	12.4±2.8	10.02.4±2.8	0.035
IL-1β (pg/ml) (mean ±sd)	9.67±4.5	4.77±4.5	<0.05	8.97±2.5	7.88±1.5	0.71
IL-6 (pg/ml) (mean ±sd)	15.65*±*5.19*	10.35*±*3.19	<0.05	16.11*±*4.23*	14.31*±*2.11	0.85
IL-10 (pg/ml) (mean ±sd)	4.25*±*2.19*	4.75*±*2.19*	<0.05	4.54*±*3.20*	4.01*±*3.20*	0.78
TNF-α (pg/ml) (mean ±sd)	13.08±3.88	8.06±2.18	<0.001	10.38±3.88	11.28±3.88	0.55
Visfatin (ng/ml) (mean ±sd)	9.21±1.54*	5.41±1.24*	<0.001	10.01*±*1.35*	9.07*±*1.35*	0.45
Leptin (ng/ml) (mean ±sd)	5.23*±*2.36	7.98.21*±*2.36	<0.05	4.23*±*2.06	4.71*±*2.06	0.39

Demographic and clinical data are expressed as percentage (n°). Laboratory variables are expressed as mean ± sd.

**HE:** hepatic encephalopathy; SBP: spontaneous bacterial peritonitis; ascites grade was evaluated by Ascites International Club criteria (5); sd: standard deviation.

**Table 3:** Comparison between the two groups treated with high dose furosemide+HSS (group A) or with seriate paracentesis. (group B) with regard of clinical and laboratory variables at discharge.

	High dose furosemide+HSS (n: 31)	Seriate paracentesis (n: 28)	p
Δ weight (Kg)	-6.02±3.8	-4.5±3.8	<0.001
Diuresis (ml/24 h)	1805±131	750±124	<0.001
Serum creatinin (mg/dl) (mean ± sd)	1.45±0.3	1.76±0.6	0.08
Sodium (mEq/L) (mean ± sd)	137±3.8	133±4.6	0.04
Potassium (mEq/L) (mean ± sd)	4.4±0.6	4.2±0.5	0.78
Urinary Na (mEq/24 h)	158 ± 25	73.5±12.4	<0.001
Urinary K (mEq/24 h)	83 ± 21	59 ± 29	0.021
Ascites at discharge (n / %)	14 (23,3)	11 (45.8)	<0.001
• Ascites grade* • Grade I • Grade II • Grade III	• • 8 (13,3) • 3 (5) • 3 (5)	• • - • 9 (37,5) • 2 (8,3)	• <0.001 • <0.001 • 0.029
Leg oedema (n / %)	4 (6.6)	10(32.2)	<0.001
Pleural effusion (n / %)	2 (3.3)	4 (16.6)	<0.001
Child Pugh score (median)	7.6	8.9	0.04
Δ Child Pugh score (median)	–1.6	- 0.9	0.012
HE (n / %)	8 (13,3)	3 (12,5)	0.67
Spontaneous bacterial peritonitis (n / %)	-	2 (8,3)	
HRS (n / %)	4 (8)	2 (8,3)	0,54
GI bleedings (n / %)	3 (5.5)	1 (4.1)	0,07
pre-existing renal failure progression (n / %)	4 (8)	2 (8,3)	0,43
Acute renal failure (n / %)	2 (4)	1 (4,1%)	0,47
Hospitalization (days) (n / %)	9.4±2.2	9.9 ±2.0	0.68
Intrahospital deaths (n/%)	2(3.3)	1 (4.4)	0,06
Δ-ANP (pg/ml)	-10.13	-2.45	<0.001
Δ-BNP (pg/ml)	-4.9	-2.38	0.031
Δ-IL-1β (pg/ml)	-4.9	-1.09	0.024
Δ-IL-6 (pg/ml)	-5.3	-1.8	0.0
Δ-IL-10 (pg/ml)	+0.50	-0.53	0.067
Δ-TNF-α (pg/ml)	-5.02	- 0.99	<0.001
Δ-visfatin (ng/ml)	-3.8	-0-94	0.039
Δ-Leptin (ng/ml)	+2.76	+0.51	0.037

Demographic and clinical data are expressed as percentage (n°). Laboratory variables are expressed as mean ± sd.

Δ weight: body weight difference (body weight at admission-body weight after treatment with high dose furosemide+HSS or seriate paracentesis); Δ Child Pugh score: Child pugh score change (Child Pugh score at admission- Child Pugh score at discharge); HE: hepatic encephalopathy; HRS: Hepathorenal Syndrome; GI bleedings: gastrointestinal bleedings; Ascites at discharge: grade of ascites valuated three days after end of diuretic treatment period or last paracentesis.

*Ascites grade was evaluated by Ascites International Club criteria (5).

Leg edema at discharge was observed in 4 (6.6%) in group A vs. 10 (32.2) in group B (p<0.001), whereas pleura effusion was observed in 2 (3.3%) subjects in Group A vs. 4 (16.6%) subjects in Group B (p<0.001). Patients in Groups A and B had similar serum levels of TNF-α, IL-1β, IL-6, ANP, BNP, visfatin and leptin at admission.

After treatment with high dose furosemide + HSS we observed a significant reduction of serum levels of TNF-α, IL-1β, IL-6, ANP, BNP, visfatin in Group A patients, whereas we observed a significant increase of leptin serum levels. In Group B we observed only a significant reduction of ANP and BNP serum levels, but no significant difference was observed regarding serum levels of TNF-α, IL-1β, IL-6 visfatin and leptin.

With regard to primary efficacy endpoints, in Group A vs. Group B we observed higher Δ-TNF-α *(-5*.*02 vs*. *- 0*.*99; p<0*.*001)*, Δ-IL-1β *(-4*.*9 vs*. *-1*.*09; p = 0*.*024)*, Δ-IL-6 *(-5*.*3 vs*. *-1*.*8; p = 0*.*067)*, Δ-ANP *(-10*.*13 vs*. *-2*.*45; p<0*.*001)*, Δ-*BNP (-4*.*9 vs*. *-2*.*38; p = 0*.*031)*, Δ-visfatin *(-3*.*8 vs*. *0*.*94; p = 0*.*039)*, Δ-Leptin *(+2*.*76 vs*. *+0*.*51; p = 0*.*037)* at discharge (Table 3)

## Discussion

In the present study we evaluated immune-inflammatory and metabolic effects of a treatment with high dose furosemide + HSS by means of analysis of changes of cytokine, natriuretic peptide, visfatin and leptin serum levels in subjects with cirrhosis and refractory ascites.

We observed a higher degree of reduction of serum levels of natriuretic peptides, inflammatory cytokines and visfatin in subjects treated with high dose furosemide plus HSS (Group A) in comparison with subjects treated with seriate paracentesis (Group B).

At discharge, patients in Group A, consistent with our own previous findings [10] had significantly higher diuresis and sodium plasma levels and significantly lower body weight, whereas patients of patients allocated to paracentesis (Group B) had a weight loss of 4.5 kg after more than 16 liters of paracentesis. This finding is due to the fact that large or refractory *ascites* frequently necessitates seriated *paracentesis and* although this procedure is effective to treat ascites, most patients with refractory ascites who underwent seriate paracentesis futherly develop ascites very early after this procedure with rapid abdominal distension and rapid weight gain thus the comulative weight loss after seriate paracentesis is relatively poor [12].

In group A subjects we also observed a significant increase in serum levels of leptin in comparison with no significant effects on serum levels of the same inflammatory and metabolic markers in Group B subjects. Thus we can conclude that treatment with high dose furosemide + HSS may modify the inflammatory and metabolic “milieu” in subjects with refractory ascites. High serum levels of some cytokines may represent a characteristic feature of cirrhosis, regardless of underlying etiology, as a direct consequence of liver dysfunction instead of an inflammatory disorder [13,14,15,16,17].

A study conducted by Kitaoka et al. [14] reported that serum levels of Th1/Th2 type cytokines are associated with progression of chronic type C liver disease. Thus, owing to the high percentage of viral cirrhosis among our recruited patients, positive effects on serum levels of some inflammatory cytokines may better explain therapeutic effectiveness of intravenous high dose furosemide plus HSS in comparison to seriate paracentesis.

Furthermore, hepatitis C infection *per se* is associated with peripheral and hepatic insulin resistance. Substrate competition by increased lipid oxidation and possibly enhanced hepatic expression of inflammatory cytokines/mediators could be involved in the defective glucose regulation [18]. No data are available with regard to the role of cytokines in the progression of other chronic liver diseases such as NASH or *alcoholic liver disease, thus our findings obtained in patients with a high prevalence of chronic HCV hepatitis are consistent with previous findings indicating a crucial role of cytokines in disease progression [19,20].*

*Inflammation also seems to have an important role on development of cirrhosis complications such as ascites and a* recent study [18] reported that in experimental cirrhosis models, an activation of the immune system occurs before ascites development.

Our findings of a significant modulation of some inflammatory and metabolic markers after treatment of refractory ascites in patients with cirrhosis may suggest that the immune-inflammatory and metabolic axis could represent a possible further pathogenic basis of cirrhosis progression and its complications. These findings have been obtained in an unselected, consecutive group of patients with refractory ascites thus limiting sampling bias and consequent biased estimates.

Genetic and environmental factors have a role in the development of some hepatic disease such as NASH and hepatic fibrosis in cirrhosis, and it has been reported that inflammatory cytokines may regulate metabolic and inflammatory changes in this context [20–22]. Furthermore, genotype-dependent differences in the development of fibrotic NASH [23] linked with immune and inflammatory responses to metabolic changes have been reported in some murine models of hepatic damage.

A recent study [24] reported that Th1-type mice on a high fat diet (HFD) regimen are more prone to adiposity, liver inflammation and fibrosis, thus confirming the strict relationship between hepatic disorders and metabolic changes.

Thus inflammatory and metabolic markers in cirrhotic subjects could represent a direct expression of fibrosis grade and severity of cirrhosis. Our findings may suggest possible speculation about the role of inflammatory and metabolic abnormalities in ascites pathogenesis.

These findings are consistent with previous findings by Pruimboom et al. [25] showing higher serum levels of some cytokines such as interleukin-1β, interleukin-6 and tumor necrosis factor-α and soluble intercellular adhesion molecule 1 in ascites and plasma samples of patients with liver cirrhosis. Our findings are also consistent with previous results by Bac et al. [26] that patients with decompensated ascites had a clear relationship between plasma interleukin-6 levels and clinical severity of cirrhosis (Child-Pugh score).

Nevertheless, to the best of our knowledge, the relationship between inflammatory markers and decompensated ascites remains an issue that has not been fully addressed. Our findings of a more significant reduction of inflammatory cytokines such as TNF-α, IL-1β, Il-6 and metabolic markers such as visfatin and leptin seem to indicate that amelioration of fluid overload in cirrhotic subjects with refractory ascites could be accompanied by parallel changes of inflammatory and metabolic pathways probably associated with an adipocite-inflammatory dysfunction that represents the metabolic substrate in subjects with decompensated liver cirrhosis.

Hypermetabolism in cirrhosis is associated with a high risk of complications and mortality as found by a recent study [27]reporting that hypermetabolic cirrhotic subjects had lower leptin serum levels.

Nevertheless in a recent study [28] authors reported a significant positive correlation between serum and ascites leptin levels. These findings indicate that in patients with decompensated liver cirrhosis, intra-abdominal production of leptin may contribute to metabolic changes, and this finding appears consistent with our findings. Another intriguing hypothesis to analyze is the possibility that HSS may express potential effects on serum levels of inflammatory cytokines and natriuretic peptides in subjects with decompensated cirrhosis.

Our group reported [12] that patients with heart failure treated with high-dose furosemide + HSS had significantly lower ANP and BNP serum levels. B-type natriuretic peptide (BNP) concentrations are higher in cirrhosis. In a study that included 83 patients hospitalized with decompensated cirrhosis. Pimenta et al. [29] observed that the BNP level in cirrhosis reflects cardiac systolic function and is an independent predictor of medium-term survival in advanced cirrhosis. Furthermore, several studies support an association between cardiac complication of cirrhosis and increased levels of natriuretic peptides with a direct relationship between natriuretic peptide serume levels and severity of liver disease [28–32].

Thus an increase of ANP and BNP serum levels seems to be strictly related to cardiac involvement in patients with cirrhosis. Nevertheless, to the best of our knowledge no study has addressed ANP and BNP changes in response to diuretic treatment in patients with cirrhosis and in patients with cirrhosis and ascites.

Several studies demonstrated that both leptin mRNA and plasma leptin levels are directly proportional to measurements of body fat [33–34]. In addition, leptin secretion is regulated by body fat-independent mechanisms. Infusion of hypertonic solutions such as galactose, mannitol, and sodium chloride have been reported to stimulate leptin secretion [34]. Thus our findings related to the increase of leptin serum levels after treatment with high dose furosemide + HSS could be related to the acute effects of intravenous HSS. Nevertheless it appears plausible that an increase in leptin levels could also be an effect of high dose furosemide in association with HSS in nutritional and metabolic patterns of cirrhotic patients

## Limitations

Our findings could be consistent with the reported role of T cells and the cytokine framework that characterize patients with HCV infection, but these findings and subsequent speculation may not represent what happens in all cirrhotic patients with chronic liver disease with other pathogenesis (alcohol, post-NASH).

Furthermore, our proposed link between treatment with high dose furosemide plus hypertonic saline solutions and an improved metabolic profile in our patients with refractory ascites could appear speculative owing to the fact that observed metabolic and inflammatory marker changes may be an epiphenomenon of the patient congestion mediated by furosemide + HSS rather than a direct effect of the therapy.

## Conclusions

We found a higher degree of reduction of serum levels of natriuretic peptides, inflammatory cytokines and visfatin in cirrhotic subjects with refractory ascites treated with high dose furosemide + HSS. We also found a significant increase in serum levels of leptin in comparison with no significant effects on serum levels of the same inflammatory and metabolic markers in subjects treated with seriated paracentesis. These findings underline possible inflammatory and metabolic effects of saline overload correction in the treatment of cirrhosis complications such as refractory ascites. This possibly suggests a role of inflammatory and metabolic-nutritional variables as severity markers in these patients.

## Supporting Information

S1 File Original Protocol Italian. Effetti immunoinfiammatori e metabolic della terapia con elevate dosi di furosemide endovena in piccoli volume di soluzione salina ipertonica in soggettti con cirrosi epatica ed ascite refrattaria.
(PDF)

S2 File Trial study protocol. Clinical Trial: Immune-inflammatory and Metabolic Effects of High Dose Furosemide Plus Hypertonic Saline Solution (HSS) Treatment in Cirrhotic Subjects With Refractory Ascite.
(PDF)

S3 File CONSORT 2010 checklist of information to include when reporting a randomised trial. Immune-inflammatory and Metabolic Effects of High Dose Furosemide Plus Hypertonic Saline Solution (HSS) Treatment in Cirrhotic Subjects With Refractory Ascite.
(DOC)
