# Supplementary material for: Immune-Inflammatory and Metabolic Effects of High Dose Furosemide plus Hypertonic Saline Solution (HSS) Treatment in Cirrhotic Subjects with Refractory Ascites
Source: PLoS One. 2016 Dec 12;11(12):e0165443. doi: 10.1371/journal.pone.0165443 (PMC5152809; doi:10.1371/journal.pone.0165443)
Supplement: S1 File — Effetti immunoinfiammatori e metabolic della terapia con elevate dosi di furosemide endovena in piccoli volume di soluzione salina ipertonica in soggettti con cirrosi epatica ed ascite refrattaria. (PDF) [file pone.0165443.s001.pdf]

Effetti immunoinfiammatori e metabolic della terapia con elevate dosi di furosemide endovena in piccoli volume di soluzione salina ipertonica in soggettti con cirrosi epatica ed ascite refrattaria

Background scientifico ed obiettivi del progetto di studio

Cirrosi e insufficienza cardiaca congestizia (CHF) sono i principali stati di malattia clinica caratterizzata da sodio renale e ritenzione di acqua con la formazione di edema. Anomalie di omeostasi circolatoria e volume in queste malattie suscitano risposte neuro-ormonali che influenzano la funzione renale e che portano alla ritenzione di sodio e acqua.

Le citochine costituiscono una rete complessa di molecole coinvolte nella regolazione della risposta infiammatoria e l'omeostasi di funzioni degli organi. Inoltre citochine coordinano processi fisiologici e patologici nel fegato, come la crescita e la rigenerazione del fegato, così come i processi infiammatori compreso malattia virale del fegato, fibrosi epatica e cirrosi (1).

Inoltre, i pazienti con malattie epatiche croniche sono solitamente sottili a causa di ipermetabolismo, l'assunzione di cibo diminuita, e la malnutrizione, e la leptina è pensato per essere coinvolti in questo processo (2).

Adipocitochine svolgono un ruolo importante nel metabolismo lipidico e la progressione della malattia epatica. Visfatina una proteina di 52 kDa che è stato clonato come fattore delle cellule pre-B colonia di miglioramento (PBEF), e nel fegato e nei muscoli sono stati segnalati per essere i tessuti con i più alti livelli di espressione di questa proteina (3). Recentemente, la visfatina è stata anche proposta come una possibile adipochina secreta dal tessuto adiposo (4).

Un recente studio (5) ha mostrato come i pazienti con infezione cronica da HBV mostrino livelli sierici significativamente più elevati di adiponectina e visfatina, ma bassi livelli di leptina rispetto ai controlli sani, e che i livelli sierici di adipocitochinico correlano in modo indipendente con HBV viremia, i livelli di HBsAg, e le fasi di fibrosi epatica.

Un altro recente studio (6) hanno riportato che in soggetti con cirrosi alcolica dopo aggiustamento

per massa grassa, livelli visfatina erano significativamente più alti di Child-Pugh Classe A alla Classe C.

Inoltre, leptina, il primo descritto adipochine, interazioni con metabolismo epatico e dati da un piccolo studio suggeriscono che la somministrazione leptina ricombinante ha un effetto eventualmente benefico sulla steatosi, ma non la fibrosi, in pazienti con NAFLD con hypoleptinemia (7) e un altro molto recente studio (8) ha riferito che in steatoepatite non alcolica (NASH), la leptina è upregulated, e promuove la fibrosi epatica attivando direttamente le cellule stellate epatiche (HSC) attraverso la via riccio e l'osteopontina riccio-regolato (OPN). Tuttavia, per quanto di nostra conoscenza nessuno studio ha affrontato l'efficacia del trattamento delle complicanze della cirrosi, come ascite e sovraccarico di liquidi su queste anomalie infiammatorie e metaboliche .

Secondo l'ascite Club International, (9) ascite refrattaria è definita dalla mancanza di risposta alle alte dosi di diuretici (spironolattone 400mg/day e 160mg/day furosemide) o lo sviluppo di effetti avversi (iperkaliemia, iponatriemia, encefalopatia epatica o renale guasto) che vietano ulteriore uso di diuretici.

Il nostro gruppo in un recente studio clinico ha riportato (10) che per via endovenosa soluzioni saline ipertoniche (HSS) più alte dosi di furosemide è una sicura ed efficace alternativa alle ripetute paracentesi nel trattamento di pazienti ricoverati con cirrosi e ascite refrattaria. Inoltre, il nostro gruppo (11) ha valutato gli effetti di furosemide ad alte dosi più soluzioni ipertoniche saline di piccolo volume (HSS) sui peptidi natriuretici e dei livelli dei marker immuno-infiammatori per analizzare la risposta di soluzione salina carico acuta dopo il trattamento, la segnalazione che il trattamento con HSS potrebbe essere responsabile di uno “stretch relief” che potrebbe influenzare i livelli sierici dei marcatori natriureitic ed immunoinfiammatori.

Obiettivi del Progetto

Su questa base, l'ipotesi del nostro studio è basato sulla ipotesi secondo cui la efficacia clinica di elevate dosi di furosemide + HSS potrebbe essere realizzato attraverso effetti paralleli sui pathway di tipo infiammatorio, natriuretico e metabolico espressi come variazione dei livelli sierici di marker di tipo infiammatorio, natriuretico e metabolici (citochine, peptidi natriuretici, leptina e visfatina) evidenziabili dopo un ciclo di terapia endovenosa con elevate dosi di furosemide+ piccoli volumi di HSS

Pertnato , come estensione del nostro precedente studio clinico, valuteremo gli effetti metabolici e infiammatori della terapia endovenosa con elevate dosi furosemide più HSS in confronto alle paracentesi ripeture + trattamento diuretico orale standard nei pazienti con cirrosi ed ascite refrattaria , valutando i possibili effetti su un panel di biomarcatori sierici come alcune citochine infiammatorie, ANP / BNP, leptina e livelli sierici visfatina mediante analisi delle differenze dei loro livelli sierici prima e dopo il trattamento con alte dosi furosemide + HSS.

Materiali e metodi

Verranno arruolati tutti i pazienti consecutivi con ascite non responsiva al trattamento afferenti presso l’ambulatorio di Epatoliogia della Azienda Ospedaliera Policlinico 'Paolo Giaccone di Palermo ricoverati presso la U.O .di Medicina Interna della Azienda Ospedaliera Policlinico “P. Giaccone” di Palermo, dal dicembre 2010 al dicembre 2014

La ascite refrattaria verrà definita secondo i criteri internazionali ascite Club 1 come: (a) *ascite refrattaria diuretico resistente*: <1.5kg /week perdita di peso durante il trattamento con furosemide (160mg/day) e spironolattone (400mg/day) ; o (b) *ascite refrattaria diuretico intrattabile*: <perdita di peso 1.5kg/week a causa della impossibilità di utilizzare una dose efficace di diuretico a causa dello sviluppo di iponatriemia indotta da diuretici (livello di sodio <125mEq/ L), iperkaliemia (potassio livello> 5.5mEq/L), insufficienza renale (raddoppio della creatinina sierica o dei valori> 2.5g/dL) o encefalopatia; (C) precedente ridotto apporto alimentare di sodio tra 50-66mEq /day.

I criteri di esclusione saranno: l'incapacità di ottenere il consenso informato, possibili ascite non cirrotica, insufficienza cardiaca congestizia (definita da esame clinico e ecocardiogramma),

insufficienza renale acuta, carcinoma epatocellulare in base alla Barcelona Clinic cancro al fegato (BCLC) criteri (13), la sepsi attivo, tumori maligni.

Un consenso informato scritto verrà ottenuto per tutti i pazienti arruolati.

Verrà eseguita una valutazione clinica e di laboratorio a cadenza quotidiana.

I soggetti arruolati verranno randomizzati rispettivamente ai seguenti gruppi di trattamento: Gruppo A: il trattamento con infusione endovenosa di furosemide (dosi 125-250mg/ bid) più piccoli volumi di HSS (150mL 1,4-4,6% NaCl), dal primo giorno dopo il ricovero fino a 3 giorni prima della dimissione, con la limitazione acqua e un normale di sodio dieta. Gruppo B: ripetuto paracentesi (4-6L al giorno) dal primo giorno dopo il ricovero entro 3 giorni dalla dimissione con albumina reinfusione ad un tasso di L 5-8g/ di ascite rimossi. L'ultima paracentesi (a 3 giorni dal ricovero) è stato un paracentesi totale (8.1 ± 2.7L) più iv albumina infusione (8 g per litro di liquido ascitico rimosso) a seguito di un metodo precedentemente descritto.

Indagini di Laboratorio

I campioni di sangue da ogni soggetto arruolato verranno raccolti dopo almeno 30 minuti di riposo a letto in posizione supina, entro 24 ore dalla ammissione e dopo 8 giorni di trattamento attivo. I campioni di sangue verranno centrifugati (10.000 g) e il supernatante risultante verrà immediatamente congelato a -80 ° C fino al momento dell'analisi. Verranno valutati i livelli sierici di ANP, BNP, leptina, visfatina, e IL-1β, TNF-a, IL-6 sono stati misurati utilizzando un sandwich ELISA (Human IL-1β, TNF-a, IL-6 6 Diaclone). Come endpoint primario di efficacia abbiamo scelto di valutare la differenza (Δ) tra valore ammissione e valore al momento della dimissione di alcune variabili di laboratorio.

• Δ-ANP (pg / ml): valutata mediante la differenza tra i livelli plasmatici di ANP a livelli d'ingresso e al plasma ANP al momento della dimissione.

• Δ-BNP (pg / ml): valutata mediante la differenza tra i livelli plasmatici di BNP a livelli plasmatici di ammissione e BNP alla dimissione.

• Δ IL-1β (pg / ml): valutata mediante la differenza tra i livelli plasmatici di IL-1β all'ammissione e livelli plasmatici di IL-1β allo scarico.

• Δ-visfatina (ng / ml): valutata mediante la differenza tra visfatina siero al momento del ricovero e visfatina siero al momento della dimissione.

• Δ-leptina (ng / ml): valutata mediante la differenza tra leptina nel siero a ammissione e leptina nel siero a scarico.

Analisi statistica

I risultati verranno presentati come media ± SD. L'analisi dei dati verranno eseguite utilizzando il test t di Student spaiato e il test non parametrico di Mann-Whitney. Il test Chi2 è stato utilizzato per confrontare le differenze efficacia endpoint tra casi e controlli. Tutti i valori P riportati meno di 0,05 sono stati considerati statisticamente significativi. Per calcolare il numero dei pazienti da arruolati, abbiamo definito efficacia significativa una differenza significativa nella metabolica e variabili immuno-infiammatorie ≥20 a scarica tra i due gruppi, con un errore beta di 20% e una potenza di 0,80. Abbiamo aggiunto altre 7 pazienti per la dimensione del campione stimato di 40 pazienti per compensare eventuali drop out; il campione finale, quindi, comprende 47 pazienti. risultati
