# Supplementary material for: Immune-Inflammatory and Metabolic Effects of High Dose Furosemide plus Hypertonic Saline Solution (HSS) Treatment in Cirrhotic Subjects with Refractory Ascites
Source: PLoS One. 2016 Dec 12;11(12):e0165443. doi: 10.1371/journal.pone.0165443 (PMC5152809; doi:10.1371/journal.pone.0165443)
Supplement: S2 File — Clinical Trial: Immune-inflammatory and Metabolic Effects of High Dose Furosemide Plus Hypertonic Saline Solution (HSS) Treatment in Cirrhotic Subjects With Refractory Ascite. (PDF) [file pone.0165443.s002.pdf]

Clinical Trial: Immune-inflammatory and Metabolic Effects of High Dose Furosemide Plus Hypertonic Saline Solution (HSS) Treatment in Cirrhotic Subjects With Refractory Ascite

Version that was submitted to and approved AOUP "P. Giaccone" ethics committee before the trial began (November 2013.

Background

Cirrhosis and congestive heart failure (CHF) are major clinical disease states characterized by renal sodium and water retention with edema formation. Abnormalities of circulatory and volume homeostasis in these diseases elicit neuro-hormonal responses influencing renal function and leading to retention of sodium and water.Cytokines constitute a complex network of molecules involved in the regulation of the inflammatory response and the homeostasis of organ functions. Moreover cytokines coordinate physiologic and pathologic processes in the liver, such as liver growth and regeneration, as well as inflammatory processes including viral liver disease, liver fibrosis and cirrhosis .

Furthermore, patients with chronic liver diseases are usually thin as a result of hypermetabolism, diminished food intake, and malnutrition, and leptin is thought to be involved in this process. Furthermore, other adipocytokines play an important role in lipid metabolism and liver disease progression. Visfatin a 52-kDa protein that has been cloned as pre-B cell colony-enhancing factor (PBEF), and liver and muscle have been reported to be the tissues with the highest expression levels of this protein. Recently, visfatin has also been proposed as an adipokine secreted by adipose tissue.

A recent study reported that patients with chronic HBV infection have significantly higher serum levels of adiponectin and visfatin, but lower leptin levels than healthy controls, and that serum adipocytokine levels independently correlate with HBV viremia, HBsAg levels, and liver fibrosis stages.

Another recent study reported that in subjects with alcoholic cirrhosis following adjustment for fat mass, visfatin levels were significantly higher from Child-Pugh Class A to Class C. Furthermore, leptin, the first described adipokine, interplays with hepatic metabolism, and data from a small study suggest that recombinant leptin administration has a possibly beneficial effect on steatosis, but not fibrosis, in NAFLD patients with hypo-leptinemia and a very recent study reported that in non-alcoholic steatohepatitis (NASH), leptin is upregulated, and promotes liver fibrosis by directly activating hepatic stellate cells (HSC) via the hedgehog pathway and the hedgehog-regulated osteopontin (OPN).

Nevertheless, to the best of knowledge, no study has addressed the effectiveness of treatment of complications of cirrhosis such as ascites and fluid overload on these inflammatory and metabolic abnormalities.

According to the International Ascites Club, refractory ascites is defined by the lack of response to high doses of diuretics (spironolactone 400mg⁄day and furosemide 160mg⁄day) or the development of adverse effects (hyperkalemia, hyponatremia, hepatic encephalopathy or renal failure) that prohibit further use of diuretics.

A recent clinical trial reported that intravenous hypertonic saline solutions (HSS) plus high-dose furosemide is a safe and effective alternative to repeated paracentesis when treating hospitalized patients with cirrhosis and refractory ascites.

Specific objective and hypothesis:

On this basis, the hypothesis of this trial was that the clinical effectiveness of high dose furosemide + HSS could be accomplished by parallel effects on inflammatory, natriuretic and metabolic pathways expressed by changes of cytokines, natriuretic peptides, leptin and visfatin serum levels after treatment.

Thus the aim of this trial will be to evalute the metabolic and inflammatory effects of intravenous high-dose furosemide plus HSS compared with repeated paracentesis and a standard oral diuretic

schedule, in patients with cirrhosis and refractory ascites, evaluating their effects on a panel of serum biomarkers such as some inflammatory cytokines, ANP/BNP, leptin and visfatin serum levels by means of analysis of differences of their serum levels before and after treatment with high dose furosemide + HSS.

Materials and methods All consecutive cirrhotic patients presenting with ascites unresponsive to ambulatory treatment at Palermo University Hospital (Azienda Ospedaliera Policlinico 'Paolo Giaccone') who will be admitted to the Internal Medicine Ward from December 2013 to December 2015 will be offered enrolment in the study protocol after a diagnosis of ascites had been made and all potential contraindications excluded.

Refractory ascites was defined according to the International Ascites Club criteria 1 as either: (a) diuretic-resistant refractory ascites: <1.5kg ⁄week weight loss while being treated with furosemide (160mg⁄day) and spironolactone (400mg⁄day) or an equivalent dose of a loop-acting and distal-acting diuretic; or (b) diuretic-intractable refractory ascites: <1.5kg⁄week weight loss as a result of the inability to use an effective dose of diuretic because of development of diuretic-induced hyponatremia (sodium level <125mEq⁄ L), hyperkalemia (potassium level >5.5mEq⁄L), renal failure (doubling of serum creatinine or values >2.5g⁄dL) or encephalopathy; (c) previous dietary restriction of sodium between 50-66mEq ⁄day.

Exclusion criteria were: inability to obtain informed consent, possible non-cirrhotic ascites, congestive heart failure (defined by clinical exam and echocardiogram), acute renal failure, hepatocellular carcinoma based on the Barcelona Clinic liver Cancer (BCLC) criteria , complete portal vein thrombosis, active sepsis or other incurable cancers. The study was approved by the institutional Ethics Committee and written informed consent was obtained for all patients.

Daily clinical and laboratory evaluation Treatment protocol Group A: treatment with intravenous infusion of furosemide (doses 125-250mg⁄ bid) plus small volumes of HSS (150mL 1.4-4.6%

NaCl), from the first day after admission until 3 days before discharge (8 days of treatment) , with water restriction and a normal sodium diet.

Group B: repeated paracentesis (4-6L daily) from the first day after admission until 3 days before discharge with albumin reinfusion at a rate of 5-8g⁄L of removed ascites. The last paracentesis (at 3 days from admission after 8 days was a total paracentesis (8.1±2.7L) plus iv albumin infusion (8g per liter of ascitic fluid removed) following a method previously described.

Blood sample collection Blood samples from each subject enrolled wil, be drawn after at least 30 minutes of bed rest in a supine position, within 24h of admission and after 8 days of active treatment. Blood samples were centrifuged (10,000g) and the resulting supernatant was immediately frozen at -80°C until analysis was completed.

Metabolic and immune-inflammatory biochemical evaluation Will be evaluate plasma levels of ANP, BNP, Leptin, visfatin, and IL-1β, TNF-a, IL-6 that will be measured using a sandwich ELISA (Human IL-1β, TNF-a, IL-6 6 Diaclone). ANP and BNP plasma concentration was measured in duplicate by a solid phase sandwich immune-radiometric assay for human BNP (IRMA, ANP and BNP, Shering cis bio int). The minimum detectable concentrations for the diagnostic tests are: TNF-a: 8pg/mL; IL-1β: <1pg/mL; IL-6: <0.81pg/mL; ANP: 3.1pg/mL; BNP: 5pg/mL.

Leptin and visfatin will be measured by ELISA Sandwich (leptin Mediagnost and visfatin Phoenix Pharmaceticals Inc); the minimum detectable concentration for these diagnostic teste were: leptin 0.8ng/ml; visfatin: 1.8ng/ml.

Arms

- **Experimental: intravenous furosemide**

  intravenous infusion of furosemide (doses 125-250mg⁄ bid)+Hypertonic saline solutions (150mL 1.4-4.6% NaCl), from the first day after admission until 3 days before discharge Intervention: drug: furosemide;

- **Active Comparator: seriated paracentesis**

no intervention Intervention: no drug only seriated paracentesis repeated paracentesis from the first day after admission until 3 days before discharge

Primary Outcome Measure:

- Δ-ANP

  difference between ANP serum levels at admission and ANP serum levels at discharge.

- Δ-BNP (pg/ml): evaluated by means of the difference between BNP plasma levels at admission and BNP plasma levels at discharge.

  difference between BNP serum levels at admission and BNP serum levels at discharge.

- Δ IL-1beta (pg/ml): evaluated by means of the difference between IL-1beta plasma levels at admission and IL-1beta plasma levels at discharge.

  difference between IL-1 beta serum levels at admission and IL-1 beta serum levels

- Δ-visfatin (ng/ml): evaluated by means of the difference between serum visfatin at admission and serum visfatin at discharge.

  difference between serum visfatin at admission and serum visfatin at discharge.

- Δ-Leptin (ng/ml): evaluated by means of the difference between serum leptin at admission and serum leptin at discharge.

  difference between serum leptin at admission and serum leptin at discharge.

- Δ-TNF-alfa (ng/ml): evaluated by means of the difference between serum TNF-alfa at admission and serum TNF-alfa discharge.

  difference between serum TNF-alfa at admission and serum TNF-alfa at discharge.

- Δ-IL-6 (ng/ml): evaluated by means of the difference between serum IL-6 at admission and serum IL-6 at discharge.

  difference between serum IL-6 at admission and serum Il-6 at discharge.
